# P145: Teaching concepts of hand hygiene to medical students: examining current practices across Australian medical schools

**DOI:** 10.1186/2047-2994-2-S1-P145

**Published:** 2013-06-20

**Authors:** R Kaur, H Razee, H Seale

**Affiliations:** 1 School of Public Health and Community Medicine, University of New South Wales, Sydney, Australia

## Introduction

Research conducted to date, has documented hand hygiene (HH) compliance rates for medical students ranging between 8% and 52%. While compliance rates have increased in recent years for medical students, they are still well below the ideal levels. The audit data by hand hygiene Australia indicate that currently hand hygiene of medical studnets in Australia is below 70% [1].

## Objectives

Our study aimed to examine current teaching and assessment practices used in Australian medical schools to teach students the concepts of HH.

## Methods

A cross-sectional survey was sent to medical education experts across all Australian medical schools (n= 17). The survey was made up of a mix of open and closed questions and statistical analysis was undertaken on all surveys using SPSS version 21.

## Results

Sixteen medical schools indicated that concepts of hand hygiene are taught and reinforced throughout the training program. Skills stations was reported as the most common teaching method used reported by fifteen medical schools followed by case scenarios were reported by twelve medical schools. At sixteen medical schools indicated that the HH concepts are assessed at least once during the medical training and assessment is done most commonly during OSCEs (Objectively Structured Clinical Examinations) and through clinical practical exams and competency checks. All medical schools rated their students hand hygiene compliance as high to very high. Teaching and learning of HH was considered adequate and was supported by good infrastructure. However half the participants did not consider HH as important as other medical concepts and role models were considered as important influence in reinforcing HH practices in a variety of clinical environments.

## Conclusion

Appropriate knowledge is a starting point for improving practice and for instilling the correct attitude to infection prevention. The frequency and method of teaching, as well as other measures aimed to enhance HH compliance amongst medical students, must be re-examined.

## Disclosure of interest

None declared
